# Tetanus as I Have Found It in Chester County

**Published:** 1898-04

**Authors:** W. P. Phipps

**Affiliations:** Kirkwood, Pa.


### TETANUS AS I HAVE FOUND IT IN CHESTER COUNTY.

By W. P. Phipps, V.M.D.,
KIRKWOOD, PA.

Tetanus is a spasmodic and continuous contraction of muscles, producing rigidity of the parts they supply, caused by the absorption of the products of a specific germ called the "tetanus bacilli." This organism is found in many soils, preferring those rich in potash, and multiplying only out of contact with the air, being anaërobic and growing best at a temperature of 95° F. to 100° F.

It is a rod-shaped bacillus, thickened at one end, and containing a spore. The spores are very resisting, requiring moist heat at 212° F. for five minutes to destroy them. The germ when in contact with an abraded surface, and the air becoming excluded, produces its tetanizing toxin.

Breeger has isolated four toxic ptomaïn substances from cultures of the so-called tetanus bacilli: 1. Tetanin, producing the symptoms of tetanus. 2. Teta-toxin, causing tumors, paralysis, and convulsions. 3. Muriate of toxin, producing tetanus and salivation. 4. Spasmotoxin, prostrates quickly with chlorine and tonic spasms.

The bacilli do not enter the blood, but remain in the tissues near the wound—only the ptomaïns being absorbed into the blood, acting similarly to strychnine, but the cases fail to respond to like treatment.

Tetanus is usually produced from traumatisms that admit the bacilli and of the character to exclude the air from the depth of the wound, as a puncture of the foot, flap-operation in myotomy of the tail, nicking and castration, etc.

Horses and sheep are the most susceptible, dogs the nearest immune, probably on account of their adaptedness of cleansing wounds. The disease may develop twelve days after the healing of a wound.

In the idiopathic form of tetanus there is no apparent abrasion, but may follow an internal lesion, as the virulent bacilli have been found in fresh feces, and Jennings suggested that this form of the disease may be caused by or through the irritation produced by intestinal vermies.

When the bacilli develop rapidly, and their products are rapidly

[1] Read before the annual meeting of the Pennsylvania State Veterinary Medical Association, Philadelphia, March, 1898.

absorbed, the disease is acute in form, and mortality is high, and *vice versa*.

As for the general symptom of tetanus, they are so distinct to the practitioner that they leave not the shadow of a doubt in diagnosis.

The prognoses in my limited experience have been grave, with the results personifying the prognosis.

This paper is written more in the hope of gaining knowledge than in the expectation of imparting anything new, except in the citing of three representative cases, taken from a day or more in my brief experience with this disease.

CASE I. was a gray gelding that I had furnished palliative treatment through an attack of influenza with gratifying results, and the doctor had been dispensed with, and the patient turned into the paddock during daytime to recruit. One evening the owner noticed the horse lame in the near front leg, and research resulted in finding a penetrating nail, which was withdrawn with apparent relief.

Four days later I was called and requested by the owner to cut the haw from over the sight, and thereby save the eye. He was much surprised when I explained that the case was tetanus (he would term lockjaw). As the horse was eating well, the owner doubted the diagnosis until symptoms were pronounced. The case terminated fatally in the acute form.

CASE II.—Called by farmer for ten-year-old mule, February 10, 1897, twelve miles due north. I found the animal with characteristic base-wide position, and the extensors tense; croup and cervical muscles hard, with head extended and tail elevated; nostrils dilated, and anxious, nervous expression of the countenance. Deglutition was impaired, although mastication was fairly good, and he consumed hay and fodder. Temperature 101.5° F.; pulse 48, full and strong, and strong peristaltic movement, apparently normal, but respiration labored and shallow from spasm of muscles used. Urination and defecation apparently normal in amount. Patient was in a comfortable stall at the end of the stable, stood next to mate, and, as there was no box-stall available, I permitted him to remain in his old position, and cautioned the owner to exclude light and surroundings that would tend to excite the patient. Found wound on the side of the pole, the size of a silver dollar, with history of recent healing. I curetted the same, produced profuse bleeding, and left instructions to apply twice daily to the part, with bristle-brush, Churchill's tr. iodine, diluted with the plain tr.

of iodine. I used concentrated medicine, as the fld. ext. cannabis Indica and belladonna, in small repeated doses by syringe in mouth, well back, and in powder form; I wrapped in tissue paper potassium iodide alternated with small doses of calomel, adding salines to drinking water to help regulate the excretory organs.

On account of the distance and expense involved, I saw the patient every third day. To the anxious inquiry of a fairly good nurse, I replied that if the patient progressed over the second week we might hope for a favorable termination. I made my fifth visit on February 13th, and the patient was going along quietly; appetite had flagged some, but still eating when encouraged by change of food; reclined less frequently, but had lain down during night; feces were hard and scant, but general tension of muscular system was more relaxed, with tail less erect, and I dared to hope for convalescence, but cautioned the owner that the enemy we were fighting was treacherous, and he must spare no patience to keep the mule quiet. On the sixteenth day I had expected to see my tetanus case, but received word that he was dead. On inquiry I learned that immediately after the termination of the second week the owner turned the mule into the yard so as to better judge of the progress of his improvement, and the next morning he was found as most of the subjects of tetanus terminate. I was disheartened when defeated in apparent view of success.

Case III. was a four-year-old gelding, fairly well bred. I told the owner "the truth, the whole truth, and nothing but the truth" as I had found it in this disease, that the form of medical treatment was decidedly uncertain, and I advised the anti-tetanic serum treatment, which was promptly rejected on account of the price quoted. I cut the wound out in the foot, that had been healed, according to the owner's statement, for two weeks, and advised the iodine treatment, covered by antiseptic poultices. I ordered an easily digested diet, and quartered him in a darkened box to await developments. As the owner seemed very particular about the expense, I overlooked the case; but he came for me in a week and told me how much he thought of the horse, which is generally the case—when the condition becomes critical and the owner fears loss by death it becomes more valuable in his opinion.

On visiting the patient I found tonic contraction of muscular system, apparent profuse salivation, but able and persisting in eating, although lips and tongue were stiffened and mouth would open but slightly. Pulse, temperature, and excretions were fairly good, but respiration was labored, and he had been standing two days.

I prescribed similar to Case II., and at the owner's earnest request sent for the anti-tetanic serum, which I administered the following day, it being the eighth since I first called. I gave 40 c.c. in three doses six hours apart, injected hypodermatically with antiseptic precautions (the immunizing units were not given). The patient grew perceptibly worse for thirty-six hours following the injection of the serum, after which the tensity of the muscular system gradually relaxed, and in three days after crisis the horse had lain down. Serous infiltration of the dependent portions of the body and limbs responded to potassium nitrate and digitalis. In ten days withdrew all medical treatment. The animal has made a perfect recovery. Was it the serum treatment? I think it was, looking at it not as a specific but as an adjuvant to our resources to combat tetanus, and especially as a preventive agent when so used.

